# TREATMENT OF RECURRENT ANTERIOR SHOULDER DISLOCATION USING THE LATARJET TECHNIQUE

**DOI:** 10.1590/1413-785220233101e261896

**Published:** 2023-02-20

**Authors:** EDUARDO ANGELI MALAVOLTA, JORGE ANTONIO BASTOS DE SOUZA, JORGE HENRIQUE ASSUNÇÃO, MAURO EMILIO CONFORTO GRACITELLI, FERNANDO BRANDÃO DE ANDRADE E SILVA, ARNALDO AMADO FERREIRA

**Affiliations:** 1 Hospital das Clínicas, School of Medicine, Universidade de São Paulo, São Paulo, SP, Brazil.; 2 Hospital do Coração. São Paulo, SP, Brazil.

**Keywords:** Shoulder Dislocation, Joint Instability, Orthopedic Procedures, Luxação do Ombro, Instabilidade Articular, Procedimentos Ortopédicos

## Abstract

**Objective:**

To describe the functional results, recurrence rate, postoperative radiographic appearance, and complications of patients undergoing the Latarjet procedure over 24 months.

**Methods:**

Retrospective case series, including adult patients with recurrent traumatic anterior glenohumeral dislocation undergoing the Latarjet procedure. We clinically evaluated patients preoperatively by the Rowe score and at six, 12, and 24 months after the procedure. The positioning, consolidation, and resorption of the graft were analyzed by plain radiography. The recurrence rates and other complications were also described.

**Results:**

We analyzed 40 patients (41 shoulders). The Rowe score median increased from 25 before surgery to 95 at 24 months after surgery (p < 0.001). We observed graft resorption in three cases (7.3%) and consolidation in 39 (95.1%). Most grafts presented adequate placement. We observed two recurrences (4.8%), one case of dislocation and one of subluxation. Seven patients (17.1%) had a positive apprehension test. The study had no cases of infection, neuropraxia, or graft breakage.

**Conclusion:**

Latarjet surgery is a safe and effective procedure in the treatment of recurrent anterior dislocation of the shoulder. This surgery enables a statistically significant improvement according to the Rowe score, with a low number of recurrences. ***Level of Evidence IV, Case Series.***

## RESUMO

**Objetivo:**

Descrever os resultados funcionais, a taxa de recidiva, o aspecto radiográfico pós-operatório e as complicações de pacientes submetidos ao procedimento de Latarjet ao longo de 24 meses.

**Métodos:**

Série de casos retrospectiva que inclui pacientes adultos com luxação glenoumeral recidivante anterior traumática submetidos ao procedimento de Latarjet. Avaliamos clinicamente os pacientes pela escala de Rowe pré-operatória e aos 6, 12 e 24 meses após o procedimento. O posicionamento, a consolidação e a reabsorção do enxerto foram analisados por radiografia simples. Descrevemos ainda as taxas de recidiva e as demais complicações.

**Resultados:**

Analisamos 40 pacientes (41 ombros). A mediana da escala de Rowe evoluiu de 25,0 antes da cirurgia para 95,0 passados 24 meses desde a cirurgia (p < 0,001). Foi observada reabsorção do enxerto em três casos (7,3%), e consolidação em 39 (95,1%). A maioria dos enxertos apresentava posicionamento adequado. Ocorreram duas recidivas (4,8%), sendo um caso de luxação e outro de subluxação. Sete pacientes (17,1%) referiam sensação de apreensão. Não ocorreram casos de infecção, neuropraxia ou quebra do enxerto.

**Conclusão:**

A cirurgia de Latarjet é um procedimento seguro e eficaz no tratamento da luxação anterior recidivante do ombro, possibilitando melhora funcional significativa de acordo com a escala de Rowe, com baixo número de recidivas. **Nível de Evidência IV, Série de Casos.**

## INTRODUCTION

The shoulder is the most common joint to dislocate,^1^ and the treatment of traumatic anterior recurrent dislocations is preferably surgical.^2^ In the presence of significant bone loss of the glenoid cavity or humerus, or in patients at high risk for recurrence, Bankart repair has high recurrence rates.^3^ In these cases, the techniques of Latarjet^4^ and Bristow^5^ provide better results to capsular ligament repairs,^6^ in a reliably and enduringly way in the long term.^7^ ^),(^ ^8^

The Latarjet procedure leads to consistent clinical improvement and low number of recurrences, according to a recent meta-analysis including 3,917 cases.^9^ Nationwide, some studies evaluate the effectiveness of bone blocks.^10^ ^)-(^ ^21^ Of these, only four evaluate patients with a minimum of 24 months of follow-up,^15^ ^),(^ ^17^ ^),(^ ^19^ ^),(^ ^20^ and six analyze series of 40 or more patients.^10^ ^),(^ ^12^ ^),(^ ^18^ ^)-(^ ^21^

Primarily, this study aimed to describe the clinical results of patients subjected to the Latarjet procedure, according to the Rowe score,^22^ at 24 months of follow-up. To describe the recurrence rate, the postoperative radiographic aspect of the graft, and the complications are secondary objectives.

## MATERIAL AND METHODS

A retrospective case series, with prospectively collected data, were conducted. The surgeries were performed by four physicians of the same institution, all effective members of the Sociedade Brasileira de Cirurgia do Ombro, with more than 10 years of experience. The procedures were carried out between 2013 and 2019. The study was approved by the local Ethics Committee with the number 68863417.2.0000.0068.

Skeletally mature patients with traumatic anterior glenohumeral recurrent dislocation were included in the study and subjected to the Latarjet procedure. The following indications were considered for the procedure: Instability Severity Index Score (ISIS)^23^ score with ≥ 4 score, bone loss of the glenoid cavity greater than 20%, off-track injury^24^ or recurrence after Bankart repair. All of them had clinical and pre and post operative evaluation and by image a two-year follow-up. Patients with multidirectional or posterior shoulder instability, or with a single episode of dislocation, were not included. Patients with associated rotator cuff tear or fractures other than those of the anterior rim of the glenoid cavity or of Hill-Sachs, were also not included.

### Surgical procedure and rehabilitation

The anesthesia used was the interscalene block associated with general anesthesia. The positioning used was the horizontal dorsal decubitus with the dorsum elevated at about 30°. Antibiotic prophylaxis with Cefazolin 2 g from 8 to 8 h, for a period of 24 h, was performed. The implantable materials consisted of cancellous screws or 4 mm diameter cannulated with partial thread and washers.

The surgeries were performed via deltopectoral approach. The coracoacromial ligament and the pectoralis minor muscle were disinserted from the lateral and medial face of the coracoid process, keeping the conjoint tendon intact. Using a curved osteotome, we performed osteotomy of the coracoid process near its base, sparing the coracoclavicular ligaments, obtaining a graft about 2.5-cm long. Bone spicules at the base of the graft and remaining soft tissues were removed. The lower surface of the graft was then decorticated with oscillating saw. Using a 2.5-mm drill, two holes were drilled perpendicular to the longitudinal axis of the graft, 5 to 10 mm apart. The glenoid neck was accessed by a longitudinal incision in the direction of the subscapularis fibers (split), performing the resection of the glenoid labrum and the cruentation of the bone surface. The graft was provisionally fixed to the anterior rim of the glenoid cavity with Steinmann wires. Once the correct positioning of the graft was verified (alignment with the joint surface and below the “equator” of the glenoid) with radioscopy, the neck of the glenoid cavity was drilled and the graft was fixed with two 4-mm diameter partially-threaded cancellous screws. Washers were used in all cases.

Patients used a sling for 21 days and movements for the hand, wrist, and elbow were stimulated from the first postoperative day. The passive movement arc gain was initiated at 14 days, while the active gain at 21 days. Isometric exercises were initiated at 30 days and active resisted at 45 days. Sports that used the upper limbs and manual labor were allowed between four and six months, depending on the arc of movement gain and re-establishment of strength.

### Outcomes

Rowe^22^ score was adopted as the primary outcome at 24 months postoperatively. Were considered secondary outcomes: scores by the Rowe score at six and 12 months, recurrence rate, postoperative radiographic aspect of the graft, and presence of complications.

### Variables analyzed

Clinical evaluation: Rowe score^22^ and ISIS^23^.

Factors intrinsic to the patient: age at the time of surgery, gender, dominance, smoking, epilepsy, number of previous dislocations, previous surgeries, and sports activity.

Factors related to the injury: bone loss of the glenoid cavity, Hill Sachs interval, and on-track or off-track pattern verification.

Recurrence: complete dislocation, subluxation, or positive apprehension test.

Postoperative aspect of the graft: presence of consolidation, resorption, and vertical and horizontal positioning.

Clinical complications: neurological lesions (specifically of the axillary, musculocutaneous, and suprascapular nerves), infections (superficial or deep), hematoma, and need for reoperation.

### Evaluation methods

Clinical evaluation: the clinical scores were applied by a research assistant, non-study participant, one week before the procedure, and additionally at six, 12, and 24 months for the Rowe score.

**Figure 1:**
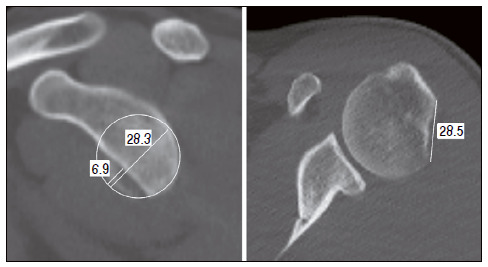
Bone loss of the glenoid cavity, measured by the best fit circle method, and of the humerus, measured by the Hill-Sachs interval.

Image evaluation: computed tomography of all patients was performed preoperatively. The measurement of the glenoid bone loss was performed by the best-fit circle,^25^ while the humerus bone loss by the Hill-Sachs interval^24^ (Figure 1). The on-track or off-track pattern verification was also performed.^24^ The postoperative evaluation of the graft, regarding to the consolidation, resorption, and positioning, was performed by simple radiographs, taken at 24 months.

### Statistical analysis

The data normality, performed by the Shapiro-Wilk test, showed that most continuous data have nonparametric distribution. Thus, continuous data were expressed as mean, standard deviation, median, and interquartile interval. Categorical data were expressed as absolute and percentage values. The Rowe score evaluation over time was performed by Friedman’s test. The comparison between sequential evaluation times was performed by the Wilcoxon test, with Bonferroni adjustment for multiple comparisons. The p ≤ 0.05 value was considered statistically significant. The SPSS program version 22.0 (SPSS Inc®, Chicago, IL, USA) was used.

## RESULTS

We performed 44 Latarjet surgeries in the evaluated period. We did not include two patients with full-thickness rotator cuff repair and one patient with multidirectional instability, in which reverse remplissage was performed together with bone block. Then, 40 patients (41 shoulders) were analyzed. Table 1 shows the general characteristics of the sample referring to patients.

**Table 1:** General characteristics of the sample (variables of patients).

Age [median (IQI)]	28	(13.0)
Male [n (%)]	39	(95.1)
Dominant side affected [n (%)]	26	(63.4)
Tobacco use [n (%)]		
Smoker	6	(14.6)
Former smoker	2	(4.9)
Number of previous dislocations [n (%)]		
2 to 5	7	(17.1)
6 to 10	5	(12.2)
11 to 20	8	(19.5)
21 to 50	10	(24.4)
> 50	11	(26.8)
Epilepsy [n (%)]	1	(2.4)
Sports activity [n (%)]	20	(48.8)
Previous Bankart repair [n (%)]	2	(4.9)

Of the 20 patients who practiced sports, ten played soccer, two rugby, two weight training, one judo, one cycling, one volleyball, and one swimming.

Patients had a median of 20% (IQR 9.2) of glenoid bone loss, and 19.6 mm (IQR 5.3) of Hill-Sachs Interval. Table 2 shows the results. A total of 29 patients (70.7%) presented an off-track lesion. The ISIS score had a median of 4.0 (IQR 2.0).

**Table 2:** Preoperative bone loss.

	Mean	sd	Median	IQI
Glenoid width (mm)	27.8	2.6	28.0	2.7
Glenoid defect (mm)	5.6	2.4	5.6	2.8
% Glenoid loss	19.9	8.2	20.0	9.2
Hill-Sachs Interval	19.3	4.9	19.6	5.3

sd: standard deviation; IQI: interquartile range.

The Rowe score evaluation showed a median of 25 before surgery and 95 at 24 months, with statistically significant changes over time (p < 0.001). Improvement occurred between the preoperative period and six months. Table 3 shows these data.

**Table 3:** Pre and postoperative functional evaluation.

	Mean	sd	Median	IQI	p*	p**
Rowe Score						
Preoperative	29.1	15.6	25.0	25.0		< 0.001
6 months	83.2	16.6	90.0	20.0	< 0.001	
12 months	85.1	16.1	95.0	22.5	0.726	
24 months	84.4	17.3	95.0	17.5	> 0.999	

* Bonferroni correction for multiple comparisons, considering sequential evaluation times; ** Friedman test; sd: standard deviation; IQI: interquartile range.

**Figure 2:**
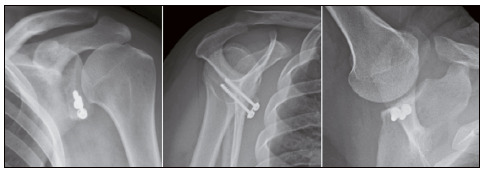
Consolidated and properly positioned graft.

**Figure 3:**
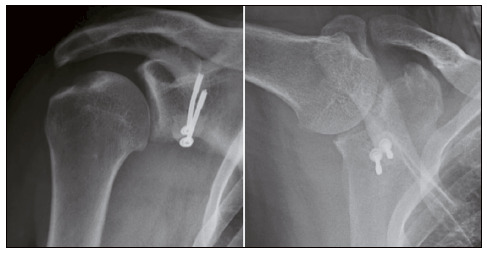
Medialized graft.

**Figure 4:**
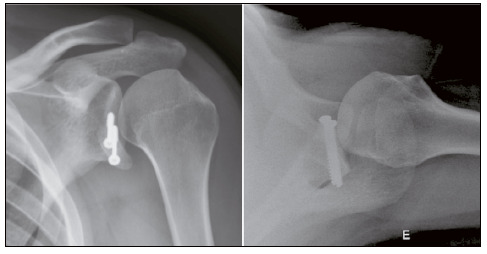
Lateralized graft.

**Figure 5:**
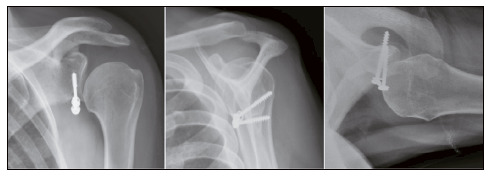
Graft with resorption.

We observed graft resorption in three cases (7.3%), and consolidation in 39 (95.1%). Most grafts presented adequate positioning. Table 4 shows these data. Figures 2, 3, 4, and 5 show, respectively, well-positioned and consolidated graft, medialized graft, lateralized graft, and graft resorption. Four patients (9.8%) had grade I glenohumeral arthrosis of Samilson and Prieto before surgery, and we observed no worsening in joint degeneration or emergence of new cases.

**Table 4:** Postoperative characteristics.

	N	%
Resorption	3	7.3
Vertical positioning		
Below the equator	40	97.6
At the equator	1	2.4
Horizontal positioning		
Well positioned	37	90.2
Medialized 2-5 mm	1	2.4
Medialized > 5 mm	2	4.9
Lateralized 2-5 mm	1	2.4
Consolidation	39	95.1

We had two recurrences (4.8%), one case of dislocation and another of subluxation. Dislocation occurred in a female patient, without traumatic event. She presented ligament laxity, although without multidirectional instability, 27.6% of glenoid bone loss, and 18.5 mm of Hill-Sachs interval. Subluxation occurred in a male patient, with 10% of glenoid bone loss and 12.1 mm of Hill-Sachs interval, after traumatic event. In both cases the graft was consolidated and well positioned, and the patients did not practice sports. Seven patients (17.1%) had a positive apprehension test when positioning the shoulder in elevation and lateral rotation. We reported no cases of infection, neuropraxia, or graft breakage.

## DISCUSSION

The Latarjet procedure led to statistically significant improvement according to Rowe score, with the median going from 25 preoperatively to 95 after 24 months. This datum is in accordance with the other authors, who report a mean Rowe from 89 to 95^11^ ^)-(^ ^13^ ^),(^ ^17^ ^),(^ ^20^ after the procedure.

We observed a recurrence rate of 4.9%, one dislocation and one subluxation, values close to that reported by meta-analysis by Gilat et al.^9^ (2.2% and 2.7%, respectively). In national studies, the recurrence rate ranges from 0%^10^ ^),(^ ^11^ ^),(^ ^13^ ^),(^ ^14^ ^),(^ ^16^ ^),(^ ^17^ ^),(^ ^19^ ^),(^ ^20^ to about 4%.^12^ ^),(^ ^15^ ^),(^ ^18^ However, these data should be analyzed with caution, since the follow-up time varies widely in studies evaluating long-term results,^15^ while others include in their sample patients with a minimum follow-up of six months.^13^ ^),(^ ^16^ ^),(^ ^21^ Moreover, because the sample of most studies is not very broad, significant percentage variations may occur, given a low level of events.

The cases also demonstrated that 17% of patients had a positive apprehension test and, although this number is similar to that pointed out by Belangero et al.,^19^ it is higher than the other national studies, which reported rates from 0%^10^ ^),(^ ^13^ ^),(^ ^14^ ^),(^ ^16^ ^),(^ ^18^ ^),(^ ^20^ to about 10%.^12^ ^),(^ ^17^ Gilat et al.,^9^ in the largest sample on the subject, recorded 2% of patients with this symptom. We believe that had a positive apprehension test is an often neglected symptom and it is not specifically evaluated by most clinical scores, except Rowe’s.^22^ ^),(^ ^26^ We do not consider this symptom a failure of the procedure, but to evaluate it in a standardized way is crucial.

The pseudarthrosis incidence in our case series was 4.9%. These values are in accordance with those reported by other authors, with rates from 0 to 12%.^10^ ^)-(^ ^14^ ^),(^ ^16^ ^),(^ ^17^ ^),(^ ^19^ ^)-(^ ^21^ Meanwhile, Ferreira Filho et al.,^18^ reported a much higher incidence, with 38% of the grafts without radiographic consolidation; however, they used single screw fixation (Bristow technique) in most cases. Graft resorption occurred in 7.3% of the cases. Other authors report rates from 0 to 15%.^10^ ^),(^ ^11^ ^),(^ ^14^ ^),(^ ^16^ ^)-(^ ^21^ Cohen et al.^17^ report 50% of resorption, but these authors perform the evaluation by computed tomography, while the others by plain radiography.

Radiographic analysis demonstrated adequate graft positioning in most cases (vertically in 98% and horizontally in 90%). These data are in accordance with the other studies, in which positioning errors are described in 3.7% to 10.5% of the samples.^13^ ^),(^ ^15^ ^)-(^ ^19^ ^),(^ ^21^ Grafts fixed too high or low may be responsible for residual instability, in the same way as excessive medial positioning. Meanwhile, lateral grafts, can lead to limitation range of motion and development of early arthrosis. Moreover, few studies evaluate the graft positioning by computed tomography.^13^ ^),(^ ^17^ ^),(^ ^21^

We reported no cases of infection in our series. The other national authors either do not describe this complication^11^ ^),(^ ^12^ ^),(^ ^14^ ^)-(^ ^18^ or report rates from 2 to 5%.^10^ ^),(^ ^13^ ^),(^ ^19^ ^)-(^ ^21^ We also did not observe any neurological lesion, as well as most national authors.^10^ ^)-(^ ^15^ ^),(^ ^17^ ^),(^ ^18^ ^),(^ ^20^ ^),(^ ^21^ Neuropraxias of the axillary or musculocutaneous nerves are mentioned by some authors.^16^ ^),(^ ^19^ ^),(^ ^21^ According to the meta-analysis by Gilat et al.,^9^ infection occurs in 0.7% of cases, and neurological injury in 0.1%.

Our study presented limitations. This is a retrospective case series, although data collection occurred prospectively, and presents the inherent biases to this type of study. The sample, although robust in national standards, limits the performance of secondary analyses. The analysis of graft positioning, consolidation, and resorption was performed by radiography, other than by computed tomography, a more accurate method used by other authors.^13^ ^),(^ ^17^ ^),(^ ^21^ Finally, the follow-up of the patients occurred for two years, a time that, superior to other authors but not ideal for assessing shoulder instability. We believe that a five-year follow-up, as done by Belangero et al.,^19^ is ideal for the evaluation of shoulder instability.

As favorable points, we highlight the 41 shoulder series, similar to the other large national series,^10^ ^),(^ ^12^ ^),(^ ^18^ ^)-(^ ^20^ with patients followed in a standardized way over two years, clinically and radiographically.

## CONCLUSION

Latarjet surgery is a safe and effective procedure in the treatment of recurrent anterior shoulder dislocation. This surgery leads to statistically significant improvement according to Rowe score, with low number of recurrences.
